# STIG study: real-world data of long-term outcomes of adults with Pompe disease under enzyme replacement therapy with alglucosidase alfa

**DOI:** 10.1007/s00415-021-10409-9

**Published:** 2021-02-05

**Authors:** Kristina Gutschmidt, Olimpia Musumeci, Jordi Díaz-Manera, Yin-Hsiu Chien, Karl Christian Knop, Stephan Wenninger, Federica Montagnese, Alessia Pugliese, Graziana Tavilla, Jorge Alonso-Pérez, Paul Wuh-Liang Hwu, Antonio Toscano, Benedikt Schoser

**Affiliations:** 1 Department of Neurology, Friedrich-Baur-Institute, Ludwig-Maximilians University Munich, Ziemssenstr. 1, 80336 Munich, Germany; 2 Department of Clinical and Experimental Medicine, University of Messina, Messina, Italy; 3 Neurology Department, Neuromuscular Diseases Unit, Hospital de La Santa Creu I Sant Pau and Biomedical Research Institute Sant Pau (IIB Sant Pau), Barcelona, Spain; 4 Centro de Investigación Biomédica en Red en Enfermedades Raras (CIBERER), Valencia, Spain; 5 John Walton Muscular Dystrophy Research Center, University of Newcastle, Newcastle, UK; 6 Department of Medical Genetics and Pediatrics, National Taiwan University Hospital, Taipei, Taiwan; 7 Neurologische Praxis Neuer Wall, Hamburg, Germany

**Keywords:** Alglucosidase alpha, Efficacy, Enzyme replacement therapy, Glycogen storage disease type 2, Long term follow-up, Pompe disease

## Abstract

**Background:**

Pompe disease is one of the few neuromuscular diseases with an approved drug therapy, which has been available since 2006. Our study aimed to determine the real-world long-term efficacy and safety of alglucosidase alfa.

**Methods:**

This multicenter retrospective study (NCT02824068) collected data from adult Pompe disease patients receiving ERT for at least 3 years. Demographics and baseline characteristics, muscle strength, lung function (FVC), walking capability (6MWT), and safety were assessed once a year. Evaluation was done on the group and individual levels, using quantitative linear models (*t *test) and general univariate linear models (ANOVA).

**Findings:**

Sixty-eight adult Pompe disease patients from four countries (Spain, Taiwan, Italy, Germany (STIG)) participated. The mean follow-up was 7.03 years ± 2.98. At group level in all outcome measures, an initial improvement followed by a secondary decline was observed. After 10 years, the 6MWT_%pred_ showed the most sustained positive effect (*p* = 0.304). The MRC_%max_ remained stable with a mild decline (*p* = 0.131), however, FVC_%pred_ deteriorated significantly (*p* < 0.001) by 14.93% over 10 years of ERT. The progression rate of FVC_%pred_ under ERT could be explained in most of the patients (83.5%) by the disease severity at baseline. Furthermore, our study shows a decline in the FVC combined with an increase in non-invasive and invasive ventilation requirements in adult Pompe disease patients over time.

**Conclusions:**

The STIG real-world study confirms an initial efficacy of ERT in the first years with a secondary sustained decline in multiple outcome measures. Further efforts are required to establish a more valid long-term monitoring and improved therapies.

**Supplementary Information:**

The online version contains supplementary material available at 10.1007/s00415-021-10409-9.

## Background

Pompe disease, also known as glycogen storage disorder type 2, is an autosomal recessive metabolic disorder caused by an enzymatic deficiency of acid alpha-glucosidase (GAA) in lysosomes [1], first described by J.C. Pompe, G. Bischoff, and W. Putschar-independently in the year 1932 [2]. There is a continuously rising number of mutations: currently there are more than 500 GAA changes causing a variable degree of GAA enzyme deficiency [3]. A prevalence of 2.4% for Pompe disease was recently identified in adults presenting with hyperCKemia and/or limb-girdle muscular weakness in a large European cohort [4]. Overall, the incidence varies between 1 to 40.000 and 1 to 200.000 depending on ethnicity and geography [5, 6], encompassing a spectrum of infantile (IOPD) to a late-onset type of Pompe disease (LOPD). Nowadays, LOPD is considered as a multisystemic disorder, mainly dominated by the characteristic proximal and axial muscle weakness with a prominent respiratory impairment [7, 8]. Patients with Pompe disease have a high risk to require a non-invasive or invasive ventilatory support during the course of their disease [9].

Pompe disease is one of the few neuromuscular diseases with an established drug therapy. Enzyme replacement therapy (ERT) with alglucosidase alfa (Myozyme) received approval in Europe in 2006. Since then, it has been the only available therapy for Pompe disease and is administered every other week at a dosage of 20 mg/kg body weight. Using this therapy has undisputedly led to a significant improvement in the outcomes of patients wit IOPD and LOPD [10, 11]. Studies of LOPD with a follow-up of less than 5 years showed after an initial improvement a secondary stabilization or decline [12–15]. Only very few studies have investigated the long-term outcomes under ERT in LOPD patients [16, 17]. Those studies reported that patients benefit from ERT for the first 3–5 years. However, after this first period, the majority of patients experienced a slow deterioration in muscle strength, walking capability, and lung function [18]. Nevertheless, without ERT, a continuous decline in muscle strength and endurance and lung function is expected [19–21].

As new and different therapies will emerge soon, it is essential to know the benefits and safety of the current standard of care ERT, and disease progression during treatment in patients with LOPD.

We, therefore, evaluated retrospective real-world data in a multicenter setting, analyzing muscle strength, walking capability and lung function in patients with LOPD receiving ERT up to 14 years.

## Methods

### Study centers and inclusion criteria

This investigator-driven study was approved by the Ethics committee of the Ludwig-Maximilians-University Munich, Germany (IRB vote 231-16) in 2016.

The study was performed at five neuromuscular centers with longstanding experience in the diagnosis and treatment of Pompe disease: (1) the Ludwig-Maximilians-University Munich, Germany (lead center), (2) the Neurology center “*Neuer Wall”* in Hamburg, Germany, (3) the Hospital de la Santa Creu i Sant Pau in Barcelona, Spain, (4) the Neurology and Neuromuscular Diseases Unit of the University of Messina, Italy and (5) the Medical Metabolic Center in Taipei, Taiwan. The originally planned participation of Argentina and Brazil could not be realized (formerly termed ATBIG study; ClinicalTrials.gov identifier: NCT02824068), so as a substitution, Spain was included leading to the new study acronym STIG. Inclusion criteria were: aged over 8 years; willing and able to provide signed informed consent; a confirmed diagnosis of Pompe disease based on a molecular genetic examination with two mutations in the *GAA* gene and/or the detection of a decreased activity of acid alpha-glucosidase; and ERT administration for at least 3 years. Exclusion criteria were: concurrent participation in another clinical study including alglucosidase alfa or other treatment; clinically significant organic disease with the exception of symptoms relating to Pompe disease, including clinically significant cardiovascular, hepatic, pulmonary, neurologic, or renal disease, or other medical condition; serious intercurrent illness; or extenuating circumstance that precludes participation in the study or potentially decreases survival. For detailed demographic characteristics of our cohort, see Table 1. Final database lock was 31 December 2019.

**Table 1:** Study cohort characteristics at baseline

Characteristic at baseline	Mean ± SD; median	*n* (%)
Follow-up duration of ERT (years)	7.03 ± 2.98; 6.50	68 (100)
Patients		68 (100)
Male		33 (48.5)
Female		35 (51.5)
Race		
Caucasian		63 (92.6)
Asian		5 (7.4)
Genotype		
GAA IVS1 splice site mutation		53 (78)
Clinical characteristics at start of ERT		
6MWT, %pred	65.54 ± 17.49; 70.00	35 (51.5)
MRC sum score proximal, %max	77.36 ± 13.17; 77.14	54 (79.4)
FVC sitting, %pred	69.70 ± 21.36; 75.00	57 (83.8)
FVC supine, l	2.22 ± 1.17; 2.20	27 (39.1)
MIP	54.52 ± 30.32; 44.00	7 (10.3)
MEP	92.89 ± 57.51; 63.00	5 (7.4)
Assistive devices		
Wheelchair dependency		3 (4.4)
Non-invasive ventilation		20 (29.4)
Invasive ventilation		3 (4.4)
Comorbidities (more than 10%)		
Morning headache		7 (10.3)
Arterial hypertension		11 (16.2)
Scoliosis		12 (17.6)
Lordosis		15 (22.1)

### Study procedures and clinical assessments

We conducted a retrospective, real-world, mult-center study in patients with LOPD to evaluate efficacy and safety of alglucosidase alfa over a long-term period. Clinical data were collected once per year as the regulatory obligations differ between the four countries. Baseline was defined as the year of ERT start [baseline (BL)]. Consecutive yearly clinical assessments included muscle strength, 6-min walk test, and lung function test.

#### Muscle strength

The modified Medical Research Council (MRC) grading scale (0–5) was used to determine skeletal muscle strength [22]. In the proximal MRC sum score, the following muscle groups were included: neck flexor, shoulder abductors, hip flexors, and hip extensors. Results are presented as a percentage of the maximum possible score (35).

#### 6-min walk test

Values of the 6-min walk test (6MWT), a measurement for functional endurance, were converted in the percentage of the predicted of normal [23]. We received the absolute/raw values from the included study centres and then calculated the % predicted for our analysis. The following formulas were used to calculate the standard value depending on the age, height and weight of the patient:

For males: (7.57 × height in cm)—(1.76 × weight kg)—(5.02 × age)—309 m = predicted distance.

For females: (2.11 × height in cm)—(2.29 × weight kg)—(5.78 × age) + 667 m = predicted distance.

#### Pulmonary function assessments

Lung function tests included measurement of forced vital capacity (FVC) in a sitting and supine position. Values of FVC_sitting_ are presented in percentage of predicted normal values, to adjust the effects of age, weight, height and gender. We received from the different centres either absolute values or %predicted.

#### Individual level

For the outcome measures MRC sum score proximal_%max_ (MRC_%max_), 6MWT_%pred_ and FVC_%pred,_ it was assessed whether the individual patient improved, deteriorated or remained stable during ERT. The following cut-off values were defined as a minimal clinically relevant change (MCRC).

For MRC sum score proximal (MRC_%max_): The agreement of three independent clinicians as an absolute change of the MRC sum score proximal of at least 2 points (> 5.70%), was considered a relevant change. As all values of MRC_%max_ except for the first year after baseline FU1), FU7, FU10, FU12 were not normally distributed, the Wilcoxon-rank-test was used comparing the mean values of MRC_%max_.

For 6MWT_%pred_: A review by Lachmann and Schoser [24] indicates that in 10 clinical studies on Pompe disease using the 6MWT, a relative change to baseline in the 6MWT was above or within the range of 5–11%, which is established for other diseases. To detect a solid clinically relevant change, we chose a cut-off value of 10% as MCRC. Patients with a relative change in walking distance of  > 10% are determined to have a clinically relevant change. For comparison of the mean values of 6MWT_%pred_ the paired, two-sided t test was used as all criteria for this measurement were fulfilled.

For FVC_%pred_: According to recent studies, FVC_%pred_ decreases in the natural untreated course of Pompe patients of about 1–4.6% within 1 year [24]. Therefore, we defined an absolute change of  > 4% as a cut-off value for categorizing patients in deteriorated, stable or improved. According to the Shapiro–Wilk test, all values of FVC_%pred_ were normal distributed.

The following additional data were recorded at baseline: age, height, weight, gender, race, genotype, age at diagnosis, assistive devices (status of wheelchair use, ventilatory support), comorbidities, cardiac diagnostics.

### Safety

To assess the safety of ERT, the following variables were gathered during treatment: infusion associated reactions, adverse events probably related to ERT, and peak titer of anti-GAA antibodies.

### Statistical analysis

For statistical analysis, SPSS statistics version 25 was used. Descriptive and explorative analysis was carried out for all demographic data and baseline characteristics as well as for safety assessments. For all metric, normally distributed outcome measures comparing mean values of two groups, a quantitative linear model with paired two-sided t-test was performed. The significance level was set at *p* < 0.05. The normal distribution was tested by Shapiro–Wilk test, respectively, and in one case by Komolgorov–Smirnov. Metric values without normal distribution were analyzed and compared by the Wilcoxon-rank test. Due to the small sample size, a statistical analysis of FVC supine, maximum inspiratory pressure (MIP) and maximum expiratory pressure (MEP) data was not appropriately powered.

Longitudinal analysis was performed by general univariate linear models and linear regression models, testing whether differences in severity of the disease and other independent variables at baseline had an impact on the course of the disease during ERT for the main outcome measures: MRC_%max_, FVC_%pred_ and 6MWT_%pred_. Cut-off points for disease severity at baseline were determined for FVC_%pred_ and 6MWT_%pred,_ each at 75%. Patients were categorized in two groups of disease severity (group 1 with high disease severity: 6MWT_%pred_ at start of ERT < 75% and/or FVC_%pred_ at start of ERT < 75%, group 2 with low disease severity: neither 6MWT_%pred_ nor FVC_%pred_ < 75%).

## Results

### Patients and baseline characteristics

We received data from 112 patients with LOPD. After supervised data analysis for data completeness and consistency, and matching with the study inclusion and exclusion criteria, we could include 68 patients for the final analysis.

**Fig. 1:**
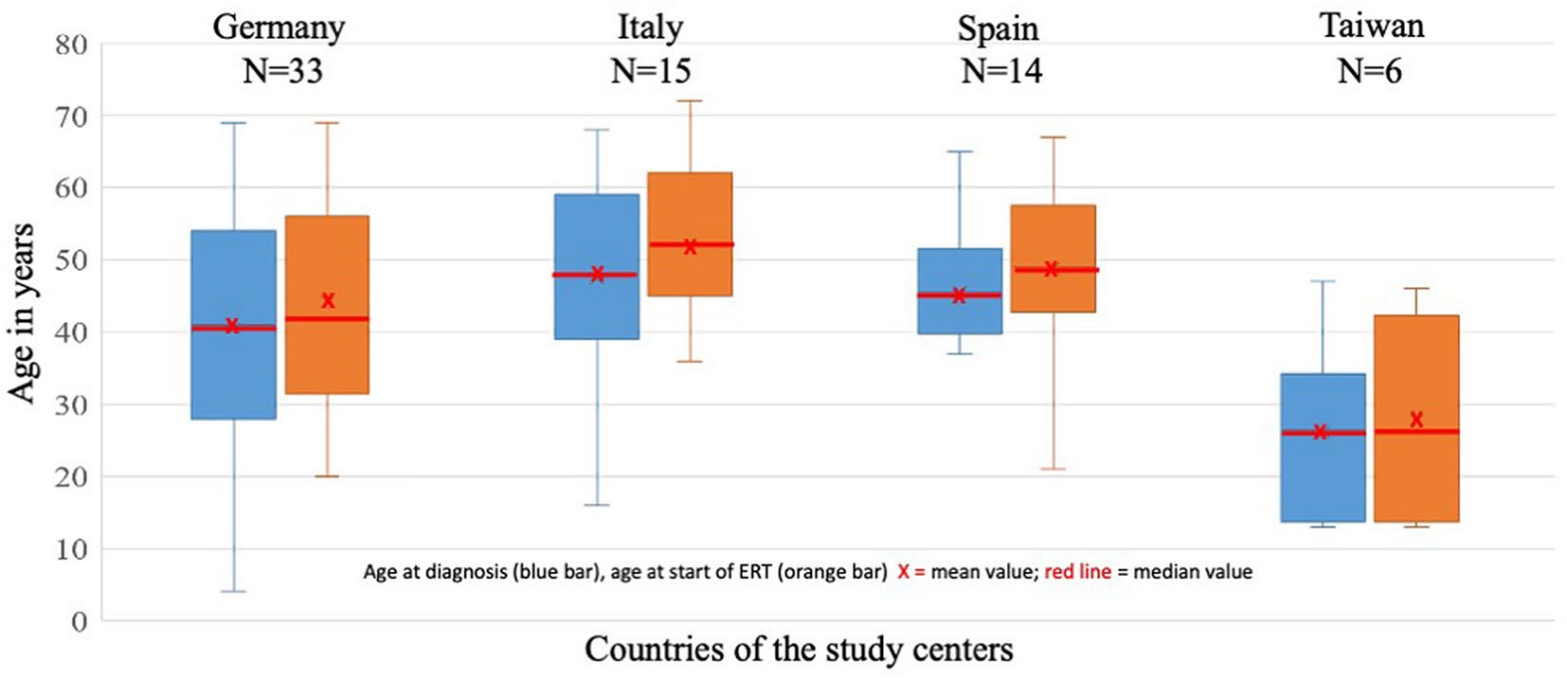
Age at diagnosis and ERT of 68 LOPD patients from the four International study centers

**Fig. 2:**
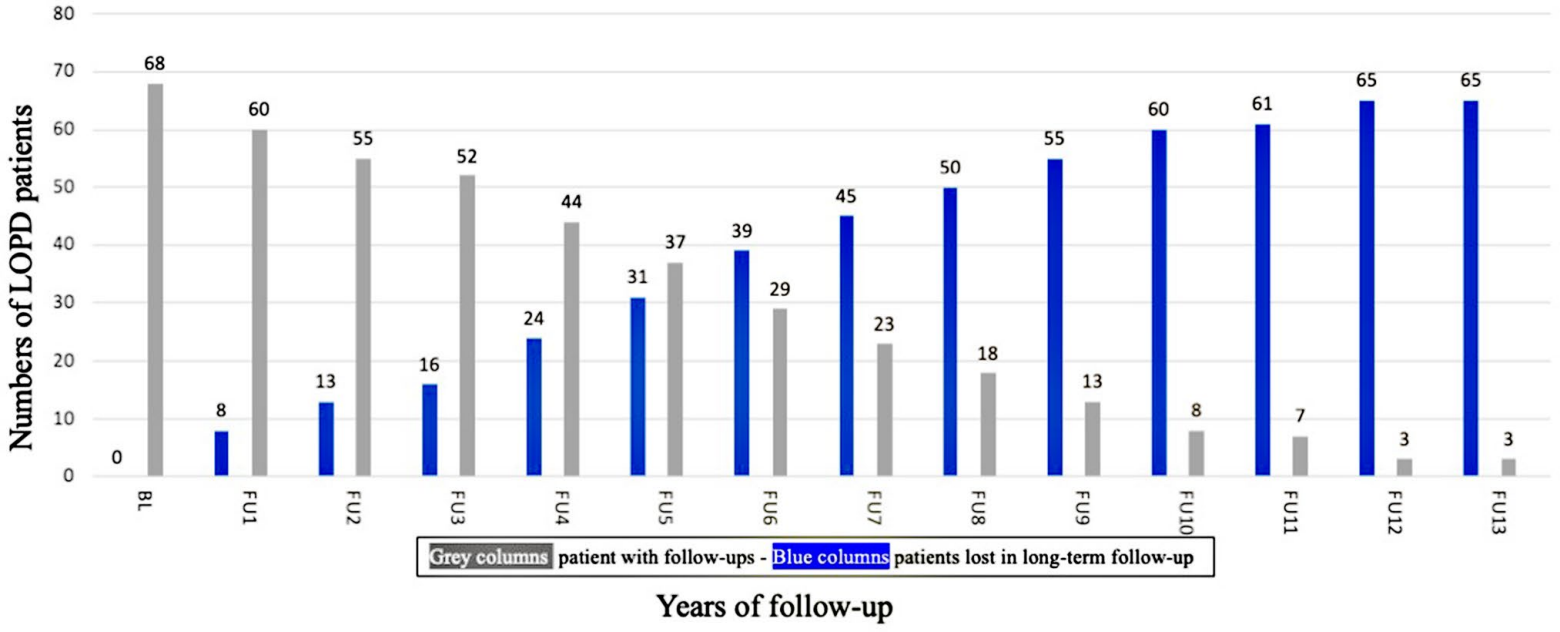
Real world data: 68 LOPD patients under ERT: lost to follow-up over 14 years

A total of 68 patients (female, 51.5%; Caucasian, 92.6%; Asian, 7.4%; Table 1) were included in the statistical data analysis, 48.5% from German centers, 22.1% from Italy, 20.6% from Spain, and 8.8% from Taiwan. The mean ± SD age at diagnosis was 41.78 years ± 15.76 (median 43.50; min 4.00; max 69.00) and the mean ± SD age at the start of ERT was 45.28 years ± 14.88 (median 45.50; min 13.00; max 72.00). The ages at diagnosis and ERT start for study center is summarized in Fig. 1. The mean ± SD follow-up duration under ERT in all study centers was 7.03 yrs. ± 2.98 (median 6.50; min 3; max 14; Fig. 2, Table 1). All had enzymatically confirmed GAA deficiency; additionally, in 85.3%, the diagnosis was confirmed by *GAA* gene analysis. The common *GAA* splice site mutation was found in 78% (Table 1).

At baseline, there were three wheelchair-dependent patients (4.4%), and ventilator support was established for 23 patients (33.8%), of which 29.4% were non-invasive, and 4.4% were invasively ventilated. Comorbidities were assessed for neuropsychological, skeletal/orthopaedic, cardiovascular, endocrine, and respiratory issues (Supplemental Table 1). At baseline, before ERT start, we noted morning headache (10.3%), arterial hypertension (16.2%), scoliosis (17.6%), and lumbar lordosis (22.1%). Further clinical characteristics at baseline are shown in Table 1.

### Muscle strength

**Fig. 3:**
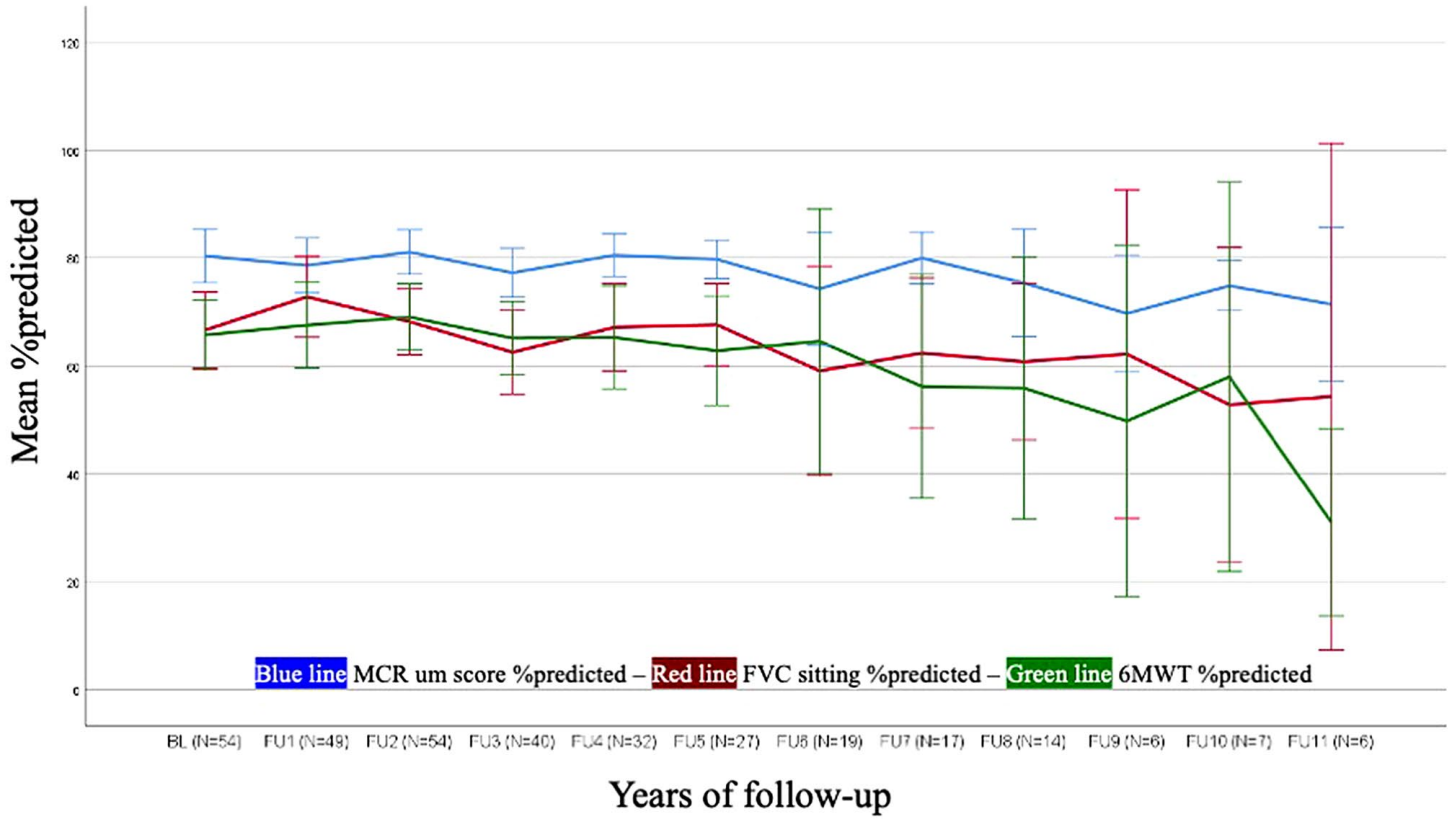
Long term follow-up of MCR_%max_, 6MWT_%pred_, FVC_%pred_ under ERT in LOPD

Data on MRC sum score was available from 54 patients at baseline. At baseline, the median value for the MRC sum score proximal in the percentage of maximum [MRC_%max_] was 77.14% (mean ± SD 77.36 ± 13.17; min 45.71; max 100.00; Table 1, Fig. 3). In the first years of ERT, the MRC score improved significantly (*p* = 0.008) by 2.66% from BL to FU1 and non-significantly (*p* = 0.171) by 1.78% from BL to FU2. There was no significant decline of MRC_%max_ between the BL and FU4 (*p* = 0.282) and FU9 (*p* = 0.131) (Supplementary Tables 2a and 2b). The longitudinal analysis revealed that disease severity at baseline has no impact on the progression rate of MRC_%max_ under ERT (*p* = 0.896).

### 6-min walk test

Data on 6MWT was available from 35 patients at baseline. At start of ERT, the median value for 6MWT_%pred_ was 70.00% (435.00 m) (mean ± SD, 65.54% ± 17.49% [436.78 m ± 133.57]; min 19% (80 m); max 103% (695 m; Table 1, Fig. 3). During ERT there was a positive effect of the 6MWT_%pred_ within the first 5 years. The increase in walking distance between BL and FU1 was 3.03% (Supplemental Table 3a). A non-significant increase of 1.93% was seen comparing the mean values of FU1 versus FU2, 0.59% for FU1 versus FU3, and 2.21% for FU1 versus FU4. In 13 patients with values for 6MWT_%pred_ at BL and FU5, there was a mean values increase of 4.00% (*p* = 0.243). At the 10-year treatment time-point, there was a non-significant decline compared to BL mean value (−16.5%; SD 26.69; *p* = 0.304) (Supplemental Table 3a and 3b). The 6MWT_%pred_ long-term data was independent of the disease severity at baseline (*p* = 0.862).

### Lung function assessments

Data on FVC was available from 57 patients at baseline. At BL, the median value for FVC_%pred_ in sitting position was 75.00% (mean ± SD 69.70 ± 21.36; min 17.00; max 125.00; Table 1, Fig. 3). Patients remained stable between BL and FU1 with a change of 2.00% (*p* = 0.237; Supplemental Table 4a), followed by a secondary decline of mean values for FVC_%pred_ in all following years by −1.82 to −21.25% (compared to FU1). The mean FVC_%pred_ decreased significantly from BL to FU4 by −3.88% (*p* = 0.045) and to FU9 by 14.93% (*p* < 0.001) (Supplemental Table 4a and 4b). The progression of FVC_%pred_ during ERT could be explained in most of the patients (83.5%) by the disease severity at baseline (*p* < 0.001). Patients with lower disease severity at baseline showed a more moderate decline of FVC_%pred_. compared to patients with higher severity of disease at baseline. For maximal inspiratory pressure (MIP) and maximal expiratory pressure (MEP), an evaluation was not appropriate due to the small number of data sets.

### Individual level

Analyzing the individual progression of patients during ERT, we compared the change of each patient with data available for FU from year to year categorizing them in three groups: improved, stabilized, deteriorated. The cut-off levels for a MCRC are explained in detail in “Methods”.

For MRC_%max_ the majority of patients remained stable from BL to FU1 and in the following years up to FU9 (Table 2). Comparing the MRC_%max_ from BL, on average, 1/3 of patients improved, 1/3 remained stable and 1/3 deteriorated overall during the following years up to FU9 (Table 2, Fig. 3).

**Table 2:** Comaprison of outcome measures categorized in deterioration, stabilization, improvement year by year

	MRC_%max_ *N* (%)				FVC_%pred_ *N* (%)				6MWT_%pred_ *N* (%)			
	*N*	Decreased	Stable	Improved	*N*	Decreased	Stable	Improved	*N*	Decreassed	Stable	Improved
BL-FU1	44	5 (11.4)	24 (54.5)	15 (34.1)	46	11 (23.9)	21 (45.7)	14 (30.4)	29	5 (17.2)	17 (58.6)	7 (24.1)
FU1-FU2	46	9 (19.6)	25 (54.3)	12 (26.1)	43	12 (27.9)	24 (55.8)	7 (16.3)	28	3 (10.7)	21 (75.0)	4 (14.3)
FU2-FU3	36	15 (41.7)	17 (47.2)	4 (11.1)	37	17 (45.9)	17 (45.9)	3 (8.1)	26	7 (26.9)	17 (65.4)	2 (7.7)
FU3-FU4	23	4 (17.4)	16 (69.6)	3 (13.0)	24	10 (41.7)	8 (33.3)	6 (25.0)	18	1 (5.6)	11 (61.1)	6 (33.3)
FU4-FU5	22	3 (13.6)	15 (68.2)	4 (18.2)	27	5 (18.5)	11 (40.7)	11 (40.7)	17	5 (29.4)	11 (64.7)	1 (5.9)
FU5-FU6	14	2 (14.3)	11 (78.6)	1 (7.1)	21	5 (23.8)	13 (61.9)	3 (14.3)	13	6 (46.2)	5 (38.5)	2 (15.4)
FU6-FU7	10	2 (20.0)	8 (80.0)	0 (0.0)	14	2 (14.3)	10 (71.4)	2 (14.3)	9	1 (11.1)	5 (55.6)	3 (33.3)
FU7-FU8	12	2 (16.7)	8 (66.7)	2 (16.7)	12	3 (25.0)	7 (58.3)	2 (16.7)	8	2 (25.0)	5 (62.5)	1 (12.5)
FU8-FU9	5	2 (40.0)	3 (60.0)	0 (0.0)	8	2 (25.0)	4 (50.0)	2 (25.0)	7	3 (42.9)	4 (57.1)	0 (0.0)
FU9-FU10	4	0 (0.0)	3 (75.0)	1 (25.0)	7	5 (71.4)	2 (28.6)	0 (0.0)	6	1 (16.7)	5 (83.3)	0 (0.0)
FU10-FU11	4	2 (50.0)	1 (25.0)	1 (25.0)	6	1 (16.7)	2 (33.3)	3 (50.0)	3	3 (100.0)	0 (0.0)	0 (0.0)
FU11-FU12	1	0 (0.0)	1 (100.0)	0 (0.0)	3	2 (66.7)	0 (0.0)	1 (33.3)	0	0 (0.0)	0 (0.0)	0 (0.0)
FU12-FU13	2	0 (0.0)	2 (100.0)	0 (0.0)	2	1 (50.0)	1 (50.0)	0 (0.0)	1	0 (0.0)	1 (100.0)	0 (0.0)

For FVC_%pred_ between BL and FU1, there was an improvement in 30% of patients, no relevant change in 46%, and deterioration in 24% (Table 2, Fig. 3).

For 6MWT_%pred_, 24% of patients improved and 59% were stable at FU1 related to BL. Over the first 6 years of FU, the majority of patients (> 60%) had stable values compared to the last year (Table 2, Fig. 3).

In the linear regression model, we did not find any significant association between the tested clinical, genetic and demographic factors across the main outcome measures (MRC_%max_, 6MWT_%pred_ and FVC_%pred_) (Table 3).

**Table 3:** Linear regression of demographic, clinical and genetic factors across the main outcome measures (MRC_%max_, 6MWT_%pred_ and FVC_%pred_)

Outcome measurement	Independent variable	Corrected *R*^2^	*p* level
Diff 6MWT mean	Gender (*N* = 39)	–0.026	0.852
	MRC at BL (*N* = 34)	–0.030	0.862
	Mutation (*N* = 37)	–0.028	0.869
Diff FVC mean	Gender (*N* = 48)	–0.010	0.461
	MRC at BL (*N* = 41)	–0.013	0.482
	Mutation (*N* = 47)	–0.017	0.637
Diff MRC mean	Gender (*N* = 51)	–0.019	0.791
	MRC at BL (*N* = 46)	–0.000	0.896
	Mutation (*N* = 49)	–0.017	0.644

Linear regression (independent variable: gender male–female, MRC_%max_ at baseline (BL), GAA IVS1 splice site mutation vs. other mutations; dependent variable: MRC_%max_, 6MWT_%pred_ and FVC_%pred_); only patients with at least two times with 2 consecutive years were considered in the analysis

### Long-term data longer than 10 years under ERT

**Fig. 4:**
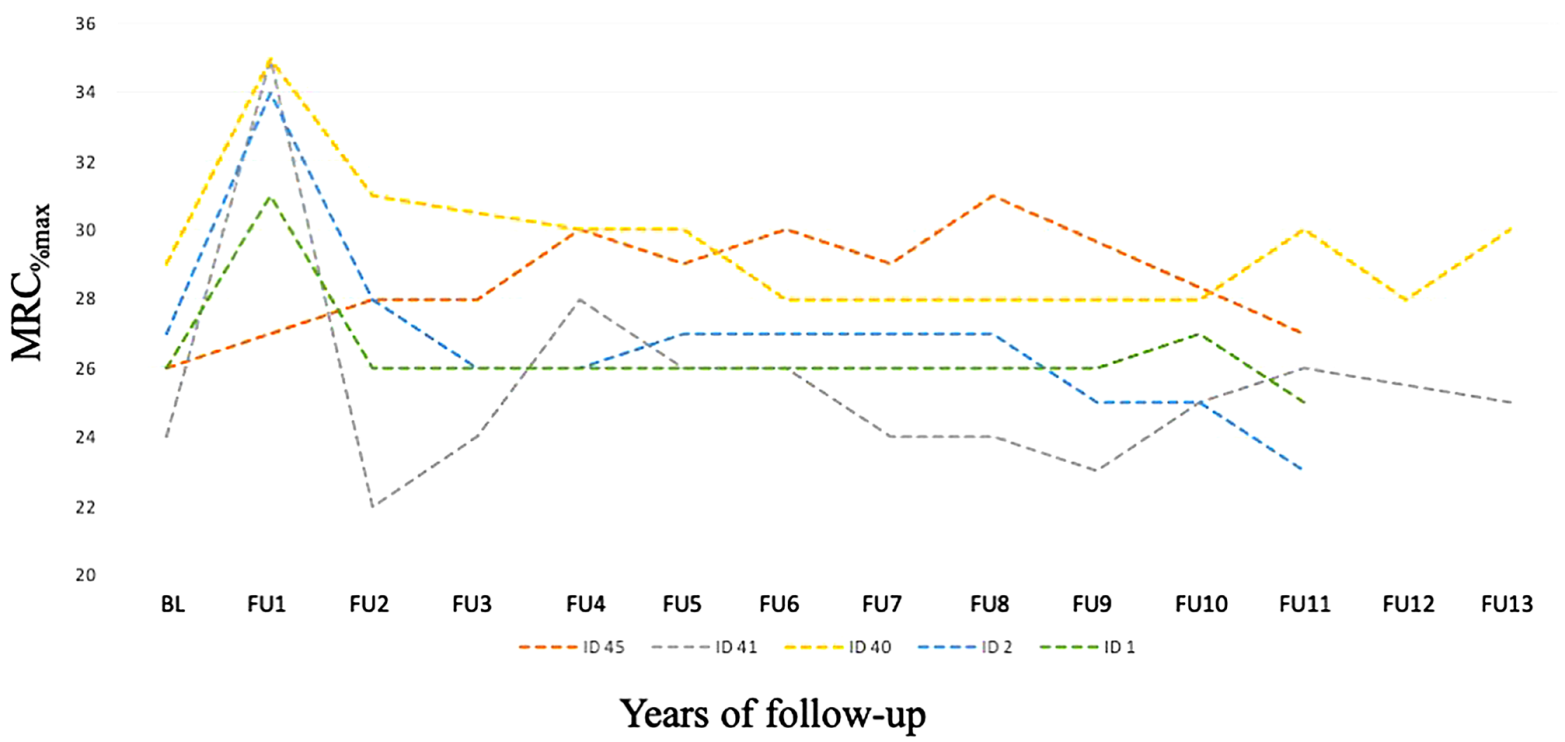
Individual MCR_%max_ scores of five LOPD patients under ERT upto 14 years

Due to our small number of patients with a follow-up on ERT of more than 10 years, a statistical analysis was of limited value. The muscle strength of five patients for up to 12 years showed no significant change in the MRC sum score. In two patients, a decline in 6MWT_%pred_, mean values from BL to FU12 was observed. In eight patients, FVC_%pred_ declined (Fig. 4, Supplemental Table 4a).

### Supportive devices

#### Ventilation status

At BL, 20 patients were non-invasively ventilated. During the observation period of up to 14 years, 18 patients started non-invasive ventilation, while invasive ventilation was initiated in three patients.

#### Wheelchair use

While at BL three patients were wheelchair dependent, nine additional patients became non-ambulatory in the course of the study.

### Long-term side effects

Serial anti-GAA antibody titers were tested in 62.3% of patients (analyses performed at Sanofi Genzyme laboratory services) and varied between 0 and 204,800, with the majority of patients (27.5%) ranging from 1:1,000 to 1:10,000, 10.1% below 1:1,000, and 8.7% above 1:10,000. There were 12 infusion-associated reactions in 9 patients, two of which led to the termination of ERT without a restart (bronchial spasm with flushing—antibody titer in the following year of 1:204,800; pulmonary embolism during infusion—antibody titer in the following year of 1:51,200). In all other cases of infusion-related events, ERT was continued—in six patients using appropriate antihistamine and/or steroidal premedication and one patient (Germany ID 043) without any known premedication. Additionally, in another six patients, side effects were classified as unrelated to ERT (Table 4).

**Table 4:** Infusion related events (IRE)

Year of FU	Type of IRE	Treatment/premedication before infusion	Study centre (pat no.)
1	Infusion related reaction (anamnestically)	n.i	Germany (053)
	Anaphylactic reaction	Antihistaminics + corticosteroids	Taiwan (022)
2	Bronchial spasms	Antihistaminics + corticosteroids	Germany (002)
	Bronchospasm with flushing; low exacerbation of an erythema with flushing (antibody titer 1:204,800 in FU2) Stop of ERT	Antihistaminics + corticosteroids	Germany (058)
	Nausea and vomiting	Antihistaminics	Germany (052)
3	Possibly anaphylactic skin reaction 05/2008	n.i	Germany (056)
	Flushing; dyspnoe, increase in blood pressure	Antihistaminics + corticosteroids	Germany (058)
	Mild anaphylactic skin reaction (pruritus at hands and feet)	Antihistaminics + corticosteroids	Germany (065)
4	Probably mild anaphylactic reaction	n.i	Germany (055)
	Proteinuria < 1 g (deposition of immune complexes due to ERT?)	n.i	Germany (043)
5	Shivering and fever during infusion	Antihistaminics + corticosteroids	Germany (055)
6	Pulmonary embolism during infusion* (antibody titer 1:51,200 in FU6) Stop of ERT	Anticoagulants	Germany (055)

## Discussion

In this real world, retrospective, long-term follow-up of 68 patients with LOPD receiving ERT, we analysed the clinical course of muscle strength, forced respiratory lung capacity, and 6-min walking capacity. The aim of the STIG Study was to evaluate the efficacy of alglucosidase alfa in a long-term period, as to date there is only limited knowledge on long-term courses over more than 5 years. Only two ERT drop-outs due to side effects of ERT were noticed in the total of 112 original screened patients with LOPD for this analysis. So, our analysis of outcomes is not biased regarding this issue. A beneficial aspect of this real-world study is the long-term ERT follow-up of 3 to up to 14 years in patients with LOPD. The use of %pred for the 6MWT and the FVC allowed a reliable comparison of values independent of gender and age.

Overall, the best therapeutic ERT effect at the group level was shown for the 6MWT. There was a sustained positive effect until FU7. Muscle strength remained stable over the years at the beginning followed by a steady decline. FVC showed the most evident decline with significant deterioration after ERT start over the course of 5 years and up to 10 years. Analogous to this pulmonary function deterioration, ventilatory support was initiated in 21 patients during the observation period.

The results of this study are in line with outcomes reported from other studies with similar or shorter follow-up periods as well as with the results of MRI studies showing a progressive increase in a fat fraction in the muscles of patients receiving ERT [25]. Positive effects are reported within the first ERT period with a secondary decline seen in the majority of patients, i.e. in the first two months [12], in the first 2–3 years [13, 14] or in the first 3–5 years, respectively [15, 18]. In our cohort, after a stable first treatment period between baseline and FU1 (2.0%, p = 0.237), there was a secondary decline of mean values in FVC_%pred_ in all the following years by −1.82 to −21.25%, which is in contrast to the results of the Late-Onset-Treatment-Study (LOTS) extension study [15, 26]. Our results are comparable with the published French Pompe registry data, describing an initial improvement in 6MWT with a cut-off point at 2.2 years and a significant improvement in motor function in the first 3 years. In the French Pompe registry study, FVC showed the weakest effect, similar to our data, by showing a decline of 0.9% per year during ERT. Another international Pompe Registry study showed a stabilization of lung function with a slope of 0.17% per year over a 5-years follow-up period [16].

On the individual level in our cohort, the MRC_%max_ compared to baseline, on average, improved in 1/3 of patients. For the 6MWT_%pred_ within the first 6 years during ERT, the majority of patients (≥ 60%) had stable or improved values compared to baseline. Long-term data beyond year 7 remain uncertain, as less than 10 patients could be analysed. Nevertheless, there was a deterioration of at least nine patients becoming non-ambulant. In the future, studies on the informative value would be improved and strengthened if a patient-reported outcome measure (PRO) is recorded in parallel to the objective test to determine the clinical relevance for the patient.

For FVC_%pred_, an overall more or less stable period up to year 6 was seen. The majority of patients relevantly decreased in their FVC_%pred_ after 6 years on ERT. As the decline of Pompe patients in FVC_%pred_ is assumed to be about 1–4.6% per year in a natural course [24], and as a deterioration in our study is defined as a change of more than 4%, the majority of our patients had still a limited benefit under ERT as without ERT therapy. Nevertheless, we found an increase of 33% from initially 1/3 non-invasive or invasive ventilated patients to 2/3 of all LOPD patients. Eighteen patients needed to start non-invasive ventilation. Whether this additional effective pulmonary therapy contributes substantially to the stabilization of LOPD patients in the late disease phase during ERT remains open.

Confounders that may also have influenced the effect during ERT compared to placebo effects, could be, in a positive sense, the motivation for physical activity and adapted nutrition, initialized by more regular monitoring or start of ventilation [27, 28]; in a negative sense, more or less pronounced comorbidities. Our results show that the disease severity at ERT start seems not to have a major impact on the overall clinical long-term course of patients under ERT, which is consistent with the latest results of a long-term study with Pompe patients under ERT from the Erasmus study group [18]. However, our STIG study reveals one important difference to former studies—our results on the FVC decline and the increase of additionally needed non-invasive and invasive ventilation requirements in the LOPD study cohort.

Overall, based on the data received, ERT was well tolerated among this long-term period of observation. Only two of 12 observed infusion associated reactions led to the stop of ERT. In addition, antibodies titers did not seem to influence the ERT response. In all other patients, ERT could be continued with appropriate antihistamine and/or steroidal premedication. Nevertheless, there should be a clear awareness of anaphylactic reactions occurring during regular ERT at any time [29]. A common reported event was fall, probably more as a result of the muscle weakness due to the disease itself in all LOPD patients instead of a side effect of ERT. Overall, there are no novel adverse events findings or infusion associated reactions to be reported.

Limitations of this study are our retrospective real-world approach so that a considerably loss of patients during the follow-up period has happened. The rising number of patients lost to follow up is the reason why only a limited comparison of the values was possible after year 7 of ERT. Another limitation, not only of this study, but rather of any Pompe disease data collection is that data on MIP and MEP are not available, as they were not collected in a clinical routine yet. An explanation for the lack of data, especially for patient-reported outcomes (PROs), is the fact that our data were collected from multiple centers and each center has a different algorithm for clinical follow-up, and at study initiation, a disease-specific PRO did not exist. Furthermore, we did not have an untreated patient cohort for statistical comparison. As this is a retrospective real-world study, there may be some variation in the performance of the tests and outcome measures, as this STIG study is not based on a standardized and controlled protocol defining in advance the methodology. However, this probable inaccuracy is unlikely to have had a major impact on data evaluation, particularly in the 6MWT or FVC, as all participating STIG centers were trained Pompe experts, used to performing standardized outcomes according to clinical trial regulatory obligations.

## Conclusion

The STIG study demonstrated that alglucosidase alfa is justifiable to be used as the first-line therapy for Pompe disease. There is an initial positive effect on the most important outcome measures, however, a more limited long-term benefit of stabilization of the clinical course under ERT for many patients over a long period. Though, according to our data, this long-term therapeutic efficacy is weakest for the lung capacity and consecutively the need of additional ventilatory support over time. As respiratory insufficiency is the most frequent cause of death in Pompe disease [21], it is important to further improve this organ function in particular. The fast approval of novel therapeutic options is a great unmet need for Pompe patients. According to initial interim evaluations, enzyme replacement therapy with the new modified drug avalglucosidase alfa appears to have a positive effect on lung capacity [30]. The clinical effect of recombinant alpha-glucosidase in the presence of a chaperone leading to improved stability of the ERT is in parallel under investigation [31]. For upcoming gene therapy trials, the safety profile of these drugs and their therapeutic impact on lung function outcomes will be a focus of interest.

Finally, based on the real-world character of the STIG study, it is highly recommended that the harmonized international standards for clinical follow-up need to be established. In addition to the frequently and regularly performed outcome measures like MRC, FVC sitting and 6MWT, especially FVC in the supine position, MIP and MEP as well as disease-specific PROs, e.g. R-PACT, and timed functional outcome measures should be part of each routine follow-up visit, at least once a year [32].

## Supplementary Information

Below is the link to the electronic supplementary material.

Supplementary file1 (DOCX 23 KB)

## Data Availability

The data that support the findings of this study are available from the corresponding author, BS, upon reasonable request.
