# Supplementary material for: STIG study: real-world data of long-term outcomes of adults with Pompe disease under enzyme replacement therapy with alglucosidase alfa
Source: J Neurol. 2021 Feb 5;268(7):2482–92. doi: 10.1007/s00415-021-10409-9 (PMC7862044; doi:10.1007/s00415-021-10409-9)
Supplement: Supplementary file 1 — Supplementary file1 (DOCX 23 KB) [file 415_2021_10409_MOESM1_ESM.docx]

# 10 Supplementary Tables

**Table 1 Comorbidities at baseline of the 68 LOPD patients**

| Comorbidities at baseline | Yes | | Missing information | | No | |
| --- | --- | --- | --- | --- | --- | --- |
|  | N | % | N | % | N | % |
| CNS disorder | 4 | 5,9% | 4 | 5,9% | 44 | 64,7% |
| Depression | 5 | 7,4% | 5 | 7,4% | 42 | 61,8% |
| Anxiety disorders | 1 | 1,5% | 1 | 1,5% | 35 | 51,5% |
| Personality disorders | 0 | 0,0% | 0 | 0,0% | 47 | 69,1% |
| Hearing loss | 4 | 5,9% | 4 | 5,9% | 45 | 66,2% |
| Symptoms of sleep disorders | 4 | 5,9% | 4 | 5,9% | 45 | 66,2% |
| Sleep disruption | 3 | 4,4% | 3 | 4,4% | 45 | 66,2% |
| Morning headache | 7 | 10,3% | 7 | 10,3% | 42 | 61,8% |
| Excessive daytime sleepiness | 4 | 5,9% | 4 | 5,9% | 46 | 67,6% |
| Polyneuropathy | 1 | 1,5% | 1 | 1,5% | 47 | 69,1% |
| Scoliosis | 12 | 17,6% | 12 | 17,6% | 36 | 52,9% |
| Kyphosis | 1 | 1,5% | 1 | 1,5% | 48 | 70,6% |
| Lordosis | 15 | 22,1% | 15 | 22,1% | 35 | 51,5% |
| Rigid spine syndrome | 0 | 0,0% | 0 | 0,0% | 49 | 72,1% |
| Arterial hypertension | 11 | 16,2% | 11 | 16,2% | 42 | 61,8% |
| Atherosclerosis | 0 | 0,0% | 0 | 0,0% | 50 | 73,5% |
| Hypercholesterolemia | 4 | 5,9% | 4 | 5,9% | 47 | 69,1% |
| Coagulopathies | 0 | 0,0% | 0 | 0,0% | 51 | 75,0% |
| Hypothyroidism | 3 | 4,4% | 3 | 4,4% | 48 | 70,6% |
| Diabetes mellitus type II | 1 | 1,5% | 1 | 1,5% | 51 | 75,0% |
| COPD-Asthma | 3 | 4,4% | 3 | 4,4% | 48 | 70,6% |
| Migraine | 2 | 2,9% | 2 | 2,9% | 49 | 72,1% |
| Chronic tension headaches | 0 | 0,0% | 0 | 0,0% | 50 | 73,5% |

**Table 2a MRC%max: Comparison of paired samples (p-value by Wilcoxon-rank test)**

|  | N | mean value - assessment 1 | mean value - assessment 2 | diff. between ass. 2 and ass.1 | standard deviation | p |
| --- | --- | --- | --- | --- | --- | --- |
| FU1_MRC%max - BL_MRC%max | 44 | 76,6891 | 79,3502 | 2,66114 | 5,69303 | 0,008 |
| FU2_MRC%max - BL_MRC%max | 48 | 78,5123 | 80,2977 | 1,78542 | 8,47059 | 0,171 |
| FU3_MRC%max - BL_MRC%max | 35 | 76,4900 | 74,5317 | -1,95829 | 7,80118 | 0,225 |
| FU4_MRC%max - BL_MRC%max | 26 | 79,4515 | 78,0219 | -1,42962 | 9,22261 | 0,282 |
| FU5_MRC%max - BL_MRC%max | 23 | 79,0074 | 77,7635 | -1,24391 | 9,66367 | 0,432 |
| FU6_MRC%max - BL_MRC%max | 17 | 73,2788 | 69,7476 | -3,53118 | 11,84640 | 0,280 |
| FU7_MRC%max - BL_MRC%max | 13 | 78,2431 | 76,2631 | -1,98000 | 9,85548 | 0,539 |
| FU8_MRC%max - BL_MRC%max | 11 | 75,5855 | 72,4673 | -3,11818 | 12,99533 | 0,483 |
| FU9_MRC%max - BL_MRC%max | 6 | 73,8100 | 70,0000 | -3,81000 | 5,90340 | 0,131 |
| FU10_MRC%max BL_MRC%max | 6 | 74,2867 | 73,3333 | -0,95333 | 4,29789 | 0,398 |
| FU11_MRC%max BL_MRC%max | 5 | 75,4300 | 74,8560 | -0,57400 | 6,82167 | 0,892 |
| FU12_MRC%max BL_MRC%max | 1^a^ | 82,8600 | 80,0000 | n.a. | n.a. | n.a. |
| FU13_MRC%max BL_MRC%max | 2 | 75,7150 | 78,5700 | 2,85500 | 0,00707 | 0,180 |

a. Correlation cannot be calculated as the sum of the cases is less than or equal to 1.

**Table 2b MAC%max: Comparison of paired samples (p-value by Wilcoxon-rank test)**

| 0 | N | mean value - assessment 1 | mean value - assessment 2 | diff. between ass. 2 and ass.1 | standard deviation | p |
| --- | --- | --- | --- | --- | --- | --- |
| comparison of paired samples (p-value by Wilcoxon-rank te+A19:G32st) | 44 | 76,6891 | 79,3502 | 2,66114 | 5,69303 | 0,008 |
| FU2_MRC%max – FU1_MRC%max | 46 | 79,2543 | 80,3728 | 1,11848 | 8,42127 | 0,398 |
| FU3_MRC%max – FU2_MRC%max | 36 | 78,4128 | 75,6358 | -2,77694 | 6,35114 | 0,016 |
| FU4_MRC%max – FU3_MRC%max | 23 | 78,2617 | 78,2609 | -0,00087 | 4,47699 | 0,824 |
| FU5_MRC%max – FU4_MRC%max | 22 | 76,2336 | 76,4923 | 0,25864 | 4,39957 | 0,975 |
| FU6_MRC%max – FU5_MRC%max | 14 | 76,5293 | 74,8971 | -1,63214 | 7,82049 | 0,651 |
| FU7_MRC%max – FU6_MRC%max | 10 | 77,4280 | 75,4280 | -2,00000 | 3,02702 | 0,068 |
| FU8_MRC%max – FU7_MRC%max | 12 | 73,8092 | 73,0950 | -0,71417 | 5,32695 | 0,588 |
| FU9_MRC%max – FU8_MRC%max | 4 | 75,0000 | 72,8575 | -2,14250 | 2,73389 | 0,180 |
| FU10_MRC%max – FU9_MRC%max | 4 | 72,8575 | 75,0000 | 2,14250 | 2,73738 | 0,180 |
| FU11_MRC%max – FU10_MRC%max | 4 | 75,0000 | 74,2850 | -0,71500 | 5,88958 | 0,581 |
| FU12_MRC%max - FU11_MRC%max | 1^a^ | 85,7100 | 80,0000 | n.a. | n.a. | n.a. |
| FU13_MRC%max - FU12_MRC%max | 1^a^ | 80,0000 | 85,7100 | n.a. | n.a. | n.a. |

a. Correlation cannot be calculated as the sum of the cases is less than or equal to 1.

**Table 3a 6MWT%predicted: Comparison of paired samples (p-value by paired t-test)**

|  | N | mean value - assessment 1 | mean value - assessment 2 | diff. between ass. 2 and ass.1 | standard deviation | p |
| --- | --- | --- | --- | --- | --- | --- |
| FU2_6MWT%pred - FU1_6MWT%pred | 29 | 65,4483 | 68,4828 | 3,03448 | 11,45639 | 0,165 |
| FU3_6MWT%pred - FU1_6MWT%pred | 27 | 66,8148 | 69,0741 | 2,25926 | 7,48636 | 0,129 |
| FU4_6MWT%pred - FU1_6MWT%pred | 21 | 64,8571 | 66,2381 | 1,38095 | 10,73069 | 0,562 |
| FU5_6MWT%pred - FU1_6MWT%pred | 13 | 60,0000 | 64,0000 | 4,00000 | 11,75443 | 0,243 |
| FU6_6MWT%pred - FU1_6MWT%pred | 15 | 62,8000 | 61,2000 | -1,60000 | 16,08371 | 0,706 |
| FU7_6MWT%pred - FU1_6MWT%pred | 9 | 63,1111 | 60,1111 | -3,00000 | 19,29378 | 0,653 |
| FU8_6MWT%pred - FU1_6MWT%pred | 6 | 65,1667 | 63,6667 | -1,50000 | 25,31995 | 0,890 |
| FU9_6MWT%pred - FU1_6MWT%pred | 6 | 68,0000 | 69,1667 | 1,16667 | 26,88060 | 0,919 |
| FU10_6MWT%pred - FU1_6MWT%pred | 4 | 66,2500 | 49,7500 | -16,50000 | 26,68957 | 0,304 |
| FU11_6MWT%pred - FU1_6MWT%pred | 3 | 68,0000 | 52,0000 | -16,00000 | 34,00000 | 0,501 |
| FU12_6MWT%pred - FU1_6MWT%pred | 2 | 66,5000 | 27,5000 | -39,00000 | 26,87006 | 0,289 |
| FU13_6MWT%pred - FU1_6MWT%pred | 0^a^ | n.a. | n.a. | n.a. | n.a. | n.a. |
| FU14_6MWT%pred - FU1_6MWT%pred | 0^a^ | n.a. | n.a. | n.a. | n.a. | n.a. |

a. Correlation cannot be calculated as the sum of the cases is less than or equal to 1.

**Table 3b 6MWT%predicted: Comparison of paired samples (p-value by paired t-test)**

|  | N | mean value - assessment 1 | mean value - assessment 2 | diff. between ass. 2 and ass.1 | standard deviation | p |
| --- | --- | --- | --- | --- | --- | --- |
| FU2_6MWT%pred FU1_6MWT%pred | 29 | 65,4483 | 68,4828 | 3,03448 | 11,45639 | 0,165 |
| FU3_6MWT%pred - FU2_6MWT%pred | 29 | 67,0000 | 68,9310 | 1,93103 | 11,53545 | 0,375 |
| FU4_6MWT%pred - FU3_6MWT%pred | 26 | 66,8462 | 63,9615 | -2,88462 | 8,89192 | 0,111 |
| FU5_6MWT%pred - FU4_6MWT%pred | 18 | 64,2778 | 68,5556 | 4,27778 | 9,55975 | 0,075 |
| FU6_6MWT%pred - FU5_6MWT%pred | 17 | 68,1765 | 63,2353 | -4,94118 | 11,05368 | 0,084 |
| FU7_6MWT%pred - FU6_6MWT%pred | 13 | 60,3846 | 56,4615 | -3,92308 | 8,84554 | 0,136 |
| FU8_6MWT%pred - FU7_6MWT%pred | 9 | 59,8889 | 61,4444 | 1,55556 | 7,38429 | 0,545 |
| FU9_6MWT%pred - FU8_6MWT%pred | 8 | 56,6250 | 54,7500 | -1,87500 | 12,13540 | 0,675 |
| FU10_6MWT%pred - FU9_6MWT%pred | 7 | 42,4286 | 39,5714 | -2,85714 | 2,19306 | 0,014 |
| FU11_6MWT%pred - FU10_6MWT%pred | 6 | 58,8333 | 56,5000 | -2,33333 | 3,38625 | 0,152 |
| FU12_6MWT%pred - FU11_6MWT%pred | 3 | 38,6667 | 31,0000 | -7,66667 | 3,51188 | 0,063 |
| FU13_6MWT%pred - FU12_6MWT%pred | 0^a^ | n.a. | n.a. | n.a. | n.a. | n.a. |
| FU14_6MWT%pred - FU13_6MWT%pred | 1^a^ | 76,0000 | 77,0000 | n.a. | n.a. | n.a. |

a. Correlation cannot be calculated as the sum of the cases is less than or equal to 1.

**Table 4a FVC&predicted: Comparison of paired samples (p-value by paired t-test)**

|  | N | mean value - assessment 1 | mean value - assessment 2 | diff. between ass. 2 and ass.1 | standard deviation | p |
| --- | --- | --- | --- | --- | --- | --- |
| FU2_FVC%pred - FU1_FVC%pred | 46 | 70,1804 | 72,1826 | 2,00217 | 11,33819 | 0,237 |
| FU3_FVC%pred - FU1_FVC%pred | 47 | 71,7660 | 70,9128 | -0,85319 | 8,99555 | 0,519 |
| FU4_FVC%pred - FU1_FVC%pred | 39 | 68,5333 | 66,2487 | -2,28462 | 10,60309 | 0,186 |
| FU5_FVC%pred - FU1_FVC%pred | 29 | 71,7793 | 67,9034 | -3,87586 | 9,96872 | 0,045 |
| FU6_FVC%pred - FU1_FVC%pred | 29 | 71,0552 | 69,1379 | -1,91724 | 10,77859 | 0,346 |
| FU7_FVC%pred - FU1_FVC%pred | 19 | 69,7368 | 65,1316 | -4,60526 | 13,06243 | 0,142 |
| FU8_FVC%pred - FU1_FVC%pred | 14 | 73,2857 | 64,9071 | -8,37857 | 8,08723 | 0,002 |
| FU9_FVC%pred - FU1_FVC%pred | 11 | 73,3636 | 62,1091 | -11,25455 | 7,54047 | 0,001 |
| FU10_FVC%pred - FU1_FVC%pred | 9 | 75,8889 | 60,9556 | -14,93333 | 7,22271 | 0,000 |
| FU11_FVC%pred - FU1_FVC%pred | 8 | 78,1250 | 55,8750 | -22,25000 | 11,84121 | 0,001 |
| FU12_FVC%pred - FU1_FVC%pred | 7 | 72,1429 | 60,4429 | -11,70000 | 8,03596 | 0,008 |
| FU13_FVC%pred - FU1_FVC%pred | 3 | 91,0000 | 76,5333 | -14,46667 | 10,96099 | 0,150 |
| FU14_FVC%pred - FU1_FVC%pred | 2 | 98,5000 | 74,9500 | -23,55000 | 14,35427 | 0,259 |

**Table 4b FVC%predicted: Comparison of paired samples (p-value by paired t-test)**

|  | N | mean value - assessment 1 | mean value - assessment 2 | diff. between ass. 2 and ass.1 | standard deviation | p |
| --- | --- | --- | --- | --- | --- | --- |
| FU2_FVC%pred - FU1_FVC%pred | 46 | 70,1804 | 72,1826 | 2,00217 | 11,33819 | 0,237 |
| FU3_FVC%pred - FU2_FVC%pred | 43 | 74,2093 | 72,3930 | -1,81628 | 10,64319 | 0,269 |
| FU4_FVC%pred - FU3_FVC%pred | 37 | 71,9595 | 68,0784 | -3,88108 | 6,23564 | 0,001 |
| FU5_FVC%pred - FU4_FVC%pred | 24 | 69,3458 | 68,8833 | -0,46250 | 7,14604 | 0,754 |
| FU6_FVC%pred - FU5_FVC%pred | 27 | 68,7926 | 70,4444 | 1,65185 | 8,44673 | 0,319 |
| FU7_FVC%pred - FU6_FVC%pred | 21 | 68,8095 | 67,9286 | -0,88095 | 4,82676 | 0,413 |
| FU8_FVC%pred - FU7_FVC%pred | 14 | 69,6786 | 69,8357 | 0,15714 | 3,28580 | 0,861 |
| FU9_FVC%pred - FU8_FVC%pred | 12 | 64,5250 | 63,3500 | -1,17500 | 7,73565 | 0,609 |
| FU10_FVC%pred - FU9_FVC%pred | 8 | 62,5000 | 61,2000 | -1,30000 | 7,77046 | 0,650 |
| FU11_FVC%pred - FU10_FVC%pred | 7 | 61,1857 | 54,4286 | -6,75714 | 5,01792 | 0,012 |
| FU12_FVC%pred - FU11_FVC%pred | 6 | 55,3333 | 62,3500 | 7,01667 | 10,53649 | 0,164 |
| FU13_FVC%pred - FU12_FVC%pred | 3 | 80,1333 | 76,5333 | -3,60000 | 7,25190 | 0,480 |
| FU14_FVC%pred - FU13_FVC%pred | 2 | 78,2500 | 74,9500 | -3,30000 | 8,06102 | 0,666 |
